# miR-197 Participates in Lipopolysaccharide-Induced Cardiomyocyte Injury by Modulating SIRT1

**DOI:** 10.1155/2022/7687154

**Published:** 2022-02-17

**Authors:** Miaomiao Liu, Ying Zhang, Xiantong Cao, Tao Shi, Yang Yan

**Affiliations:** Department of Cardiovascular Surgery, First Affiliated Hospital of Xi'an Jiaotong University, Yanta, China

## Abstract

Sepsis is a systemic inflammation and is capable of inducing myocarditis, which is a major leading cause of death in patients. Studies have found that miR-197 is correlated with the prognosis of patients with inflammatory heart disease, but its effect on sepsis-induced cardiomyocyte injury remains unclear. We treated H9c2 cells with lipopolysaccharide (LPS), then detected the cell viability via the cell counting kit-8 (CCK-8) assay and quantified miR-197 expression via quantitative real-time polymerase chain reaction (qRT-PCR). Then, we investigated the role of miR-197 in LPS-induced H9c2 cells by CCK-8 assay, flow cytometry, lactate dehydrogenase (LDH) measurement, enzyme-linked immunosorbent assay (ELISA), qRT-PCR, and western blot. Subsequently, silent information regulator 1 (SIRT1) was downregulated in H9c2 cells to explore its interaction with miR-197 under LPS induction. LPS induced miR-197 overexpression in H9c2 cells. LPS restrained viability, the expressions of B-cell lymphoma-2 (Bcl-2) and SIRT1, but promoted apoptosis, LDH release, and levels of interleukin-6 (IL-6), interleukin-1*β* (IL-1*β*), acetyl (AC)-p53, BCL2-associated X (Bax), and cleaved caspase-3 in H9c2 cells. miR-197 inhibition reversed the effects of LPS on H9c2 cells. The protective role of miR-197 downregulation in LPS-induced H9c2 cells was reversed by SIRT1 silencing. miR-197 contributed to LPS-induced cardiomyocyte injury by modulating SIRT1, which might be used as a molecular marker in the management of sepsis.

## 1. Introduction

Sepsis is a systemic inflammatory response due to microbial infection, manifested by a range of symptoms such as shortness of breath, tachycardia, fever, and abnormal white blood cell counts, which can lead to septic shock and multiorgan failure in severe cases [1–3]. Studies have revealed that when septic shock occurs, most patients are usually accompanied by septic myocarditis. Septic myocarditis is the myocardial damage caused by a large number of inflammatory factors activated in the circulatory system and released by the cardiomyocytes, which is one of the main causes of death in patients [4–7]. Despite progress in understanding of the pathology of sepsis, the molecular mechanism of the myocardial inflammation response for septic still needs to be refined [8].

With advances in molecular biology, mounting evidence has indicated that microRNA (miRNA) is a small molecule endogenous RNA that can directly act on messenger RNA (mRNA) to regulate the post-transcriptional level of target genes, playing an extremely important role in modulating cell growth, proliferation and differentiation, immune response, autophagy, and other aspects of apoptosis [9]. Besides, research has found that miRNA plays an essential role in multiple diseases, including cardiovascular diseases, where miR-197, transcribed from the 1p13.3 genomic region of the chromosome, has been proven to have a regulatory effect on many tumors and is a potential molecular marker for the diagnosis of cancer [10–12]. In recent years, some progress has been made in the study of miR-197 in cardiovascular diseases, and in particular, its abnormal expression has been shown to be involved in the occurrence and development of myocardial infarction, coronary artery disease, heart failure, and so on [13–15]. However, there are few studies focusing on the mechanism or biological function of miR-197 in sepsis-induced myocarditis.

Silent information regulator 1 (SIRT1) is a conserved NAD^+^-dependent protein deacetylase that regulates gene transcription, chromosome stability, and target protein activity through deacetylation, thereby exerting anti-inflammatory, antioxidant, and antiaging effects [16]. Available studies have demonstrated that SIRT1 can be regulated by some miRNAs and is involved in the development of diverse infectious diseases [17–19]. In the study of sepsis, the expression of SIRT1 was found to be significantly reduced in the myocardial tissue of septic mice, and activation of SIRT1 could evidently ameliorate cardiac dysfunction caused by perforation of appendix ligation [20]. However, it is poorly understood whether the effect of SIRT1 on sepsis-driven myocarditis can be regulated by miR-197.

Therefore, this study is dedicated to probing into the effect of miR-197 on sepsis-induced cardiomyocyte injury as well as elucidating its relationship with SIRT1 in order to provide new therapeutic clues for maintaining cardiac function in septic myocardial inflammation.

## 2. Methods

### 2.1. Cell Culture and Treatment

Rat cardiomyocytes H9c2 (CRL-1446) purchased from the American Type Culture Collection (ATCC, USA) were used as research subjects. Cells were grown in Dulbecco's modified Eagle's medium (DMEM, 30-2002, ATTC, USA) containing 10% fetal bovine serum (FBS, 10270106, Gibco, USA) in a humidified atmosphere at 37°C with 5% CO_2_. For the establishment of sepsis cardiomyocyte models [21], we treated H9c2 cells with 10 *μ*g/ml lipopolysaccharide (LPS, L8880, Solarbio, China) for 12 hours (h). Afterwards, the LPS-induced cells were subjected to the detection of the cell counting kit 8 (CCK-8) and quantitative real-time polymerase chain reaction (qRT-PCR) assays.

### 2.2. Cell Transfection

miR-197 inhibitor (I)/inhibitor control (IC) and small interfering RNA against SIRT1 (siSIRT1) and its negative control (siNC) were synthesized and obtained from RiboBio (Guangzhou, China). H9c2 cells were transfected with miR-197 inhibitor, inhibitor control, siSIRT1, or siNC by Lipofectamine 3000 Reagent (L3000-008, Invitrogen, Germany) following the manufacturer's protocols. After 48 h, the transfected cells were collected and subjected to LPS treatment. Subsequently, cells with different treatments were divided into the LPS group, the LPS + IC group, the LPS + I group, the LPS + I + siNC group, and the LPS + I + siSIRT1 group, and H9c2 cells without any treatment (other than normal culture) were used as the control group.

### 2.3. Cell Viability Detection

After LPS induction, the CCK-8 assay was carried out to detect the viability of H9c2 cells. The cells were washed twice with phosphate buffer saline (PBS) (C0221A, Beyotime, China) and seeded into a 96-well plate, and cultured for 24 h. Then, the CCK-8 solution (ab228554, Abcam, USA) was added to the cells and incubated at 37°C with 5% CO_2_ for another 2 h according to the operating instructions. A microplate reader (1681130-4A, Bio-Rad, China) was applied for measuring the absorbance of the cells at 450 nm.

### 2.4. Cell Apoptosis Detection

The Annexin V-FITC/Propidium iodide (PI) double staining kit (CA1020, Solarbio, China) was used in the cell apoptosis experiment. Briefly, LPS-induced H9c2 cells transfected with or without miR-197 inhibitor (1 × 10^6^ cells/ml) were harvested into a centrifugal tube (352003, Corning, USA) and centrifuged for 5 minutes at 1000 r/minutes. After being washed, the cells were suspended with 400 *μ*l 1 × binding buffer. Next, 5 *μ*l Annexin V-FITC was added into the suspension, and the cells were incubated at room temperature for 10 minutes in the dark. Afterwards, the cells were incubated with 10 *μ*l of PI for 5 minutes under the same incubation condition. Lastly, the cells were examined by flow cytometry (CytoFLEX1L5C, Beckman, USA) for apoptosis analysis.

### 2.5. QRT-PCR Assay

The expressions of miR-197 and silent information regulator 1 (SIRT1) in H9c2 cells were determined. Concretely, TRIzol reagent (15596026, Thermo Fisher, USA) or miRNeasy Mini Kit (217084, Qiagen, Germany) was used to extract total RNA from the cells following the operational manual. Next, the complementary DNA (cDNA) was synthesized using the Reverse Transcription Kit (4368814, Thermo Fisher, USA) or the Advanced miRNA cDNA Synthesis Kit (A28007, Thermo Fisher, USA). Subsequently, qRT-PCR was carried out. In a nutshell, the synthetics were reacted with PrimeScript RT Master Mix (RR036A-1, TAKARA, Japan) or TaqMan Fast Advanced Master Mix (A44360, Thermo Fisher, USA), and then developed in a Real-Time PCR system (7300, Applied Biosystems, USA). The expression levels of genes were quantified using the 2^−ΔΔCT^ approach [22]. Glyceraldehyde-3-phosphate dehydrogenase (GAPDH) and U6 were applied as the internal controls. The sequences of forward (F) and reverse (R) primers were listed as follows: miR-197 (F: 5′-ATTACTTTGCCCATATTCATTTTGA-3′; R: 5′-ATTCTAGAGGCCGAGGCGGCCGACATGT-3′); SIRT1 (F: 5′-TTAAAGCCGTGAGCCTCCAG-3′; R: 5′-ACAAAAAGCATTCCATACCGTCA-3′); GAPDH (F: 5′-AGTTAATGCCGCCCCTTACC-3′; R: 5′-CAGGGCTGACTACAAACCCA -3′); U6 (F: 5′-CGATCCAATCGGAACGGGAT-3′; R: 5′-AGGCGCCATTTCCCAACATA-3′).

### 2.6. Lactate Dehydrogenase (LDH) Analysis

The LDH assay kit (C0016, Beyotime, China) was performed to detect the content of LDH in H9c2 cells according to the protocol booklet. Before the experiment, the cells pretreated as aforementioned were planted into a 96-well plate and washed with cold PBS. 50 *μ*l of reaction mixture was added into each well, and the cells were incubated for 1 h. Then, the cells were centrifuged at 1000 rpm for 5 minutes at 4°C to remove any insoluble material. Finally, the supernatant was collected and transferred to a new plate. The absorbance at 450 nm was observed by the microplate reader to calculate the LDH level.

### 2.7. Enzyme-Linked Immunosorbent Assay (ELISA)

Levels of two proinflammatory factors interleukin-6 (IL-6) and interleukin-1*β* (IL-1*β*) in LPS-induced H9c2 cells with or without transfection of miR-197 inhibitor were determined by an ELISA. Specifically, the rat IL-1*β* ELISA kit (ab255730, Abcam, USA) was added into the cells according to the protocol booklet. Then, the cells were sealed and incubated at room temperature for 1 h. After being washed with Wash Buffer PT three times, the cells were treated with 100 *μ*l of TMB development solution for 10 minutes in the dark and with 100 *μ*l stop solution for 1 minute. Following this, the final absorbance was measured at 450 nm using the microplate reader. Ultimately, the IL-6 level was determined by the rat IL-6 ELISA kit (ab234570, Abcam, USA) following the same procedures as mentioned above.

### 2.8. Western Blot Assay

A RIPA extraction buffer (89900, Thermo Fisher, USA) was utilized to lyse total proteins in H9c2 cells. Afterwards, the BCA protein kit (ab102536, Abcam, USA) was applied for determining the concentration of total proteins. Next, protein lysates were separated by 10% SDS-PAGE sample buffer (S3401-10VL, Merck, Germany), followed by being transferred to the polyvinylidene difluoride membranes (88518, Thermo Fisher, USA). After being treated with blocking buffer (02-0866-00, Merck, Germany) for 60 minutes, the membranes were incubated with primary antibodies including those against B-cell lymphoma-2 (Bcl-2) (ab194583, 26 kDa, Abcam, USA, 1 : 500), BCL2-associated X (Bax) (ab32503, 21 kDa, Abcam, USA, 1 : 1000), cleaved caspase-3 (#9661, 17 kDa, Cell Signaling Technology, USA, 1 : 1000), SIRT1 (ab110304, 81 kDa, Abcam, USA, 1 : 1000), p53 (#2524, 53 kDa, Cell Signaling Technology, USA, 1 : 1000), acetyl (AC)-p53 (ab183544, 53 kDa, Cell Signaling Technology, 1 : 1000), and GAPDH (ab8245, 36 kDa, Abcam, USA, 1 : 1000) at 4°C overnight. Subsequently, the membranes were further incubated with secondary antibodies including those against rabbit anti-mouse IgG (ab6728, Abcam, USA) and goat anti-rabbit IgG (ab205718, Abcam, USA) at room temperature for 1 h. Later, the immunoblots were visualized using an enhanced chemiluminescence (ECL) substrate (35050, Thermo Fisher, USA) with the help of an iBright™ CL1500 Imaging System (A44240, Invitrogen, Germany). Eventually, the densitometric analyses of relative protein levels were conducted by Quantity One software (vision 4.6.6, Bio-Rad, USA).

### 2.9. Statistical Analysis

Statistical analysis was implemented using GraphPad Prism 8.0 (GraphPad Software, CA, USA). The measurement data were presented as mean ± standard deviation. Differences between the two groups were compared using an independent samples *t*-test and those among multiple groups using one-way analysis of variance (ANOVA) followed by Tukey's method. *p* < 0.05 indicated statistically significant [23].

## 3. Results

### 3.1. LPS Suppressed Viability and Induced miR-197 Overexpression in H9c2 Cells

**Figure 1:**
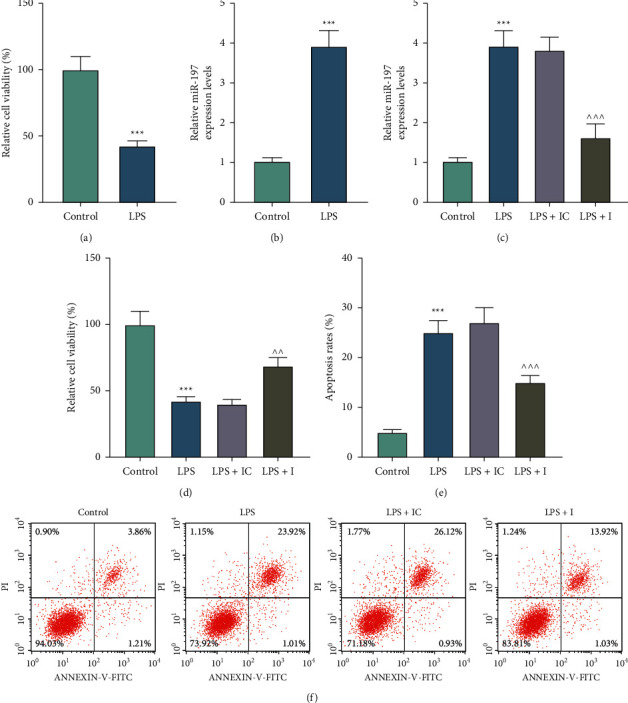
Effects of miR-197 on the viability and apoptosis of LPS-induced H9c2 cells. (a) The effect of LPS on H9c2 cell viability was detected by the CCK-8 assay. (b) The expression level of miR-197 in LPS-induced H9c2 cells was examined by qRT-PCR. U6 was used as the internal control. (c) To affirm the effect of the miR-197 inhibitor, qRT-PCR was applied to detect miR-197 expression level. U6 was used as the internal control. (d) The CCK-8 assay was conducted to examine the viability of LPS-induced H9c2 cells transfected with or without the miR-197 inhibitor. (e, f) Flow cytometry was performed to demonstrate the apoptosis of cells after LPS treatment or transfection. The experiments were repeated in triplicate. ^*∗∗∗*^*p* < 0.001 vs. control; ^∧∧^*p* < 0.01; ^∧∧∧^*p* < 0.001 vs. LPS + IC. LPS, lipopolysaccharide; CCK-8, cell counting kit-8; qRT-PCR, quantitative real-time polymerase chain reaction; I, miR-197 inhibitor; IC, inhibitor control.

The CCK-8 assay demonstrated that the viability was inhibited in H9c2 cells treated with LPS as compared with that in control cells (*p* < 0.001) (Figure 1(a)). Through qRT-PCR, we noticed that miR-197 level was upregulated in LPS-induced H9c2 cells compared with that in control cells (*p* < 0.001) (Figure 1(b)).

### 3.2. miR-197 Played a Regulatory Role in the Viability, Apoptosis, and Inflammation of LPS-Induced H9c2 Cells

**Figure 2:**
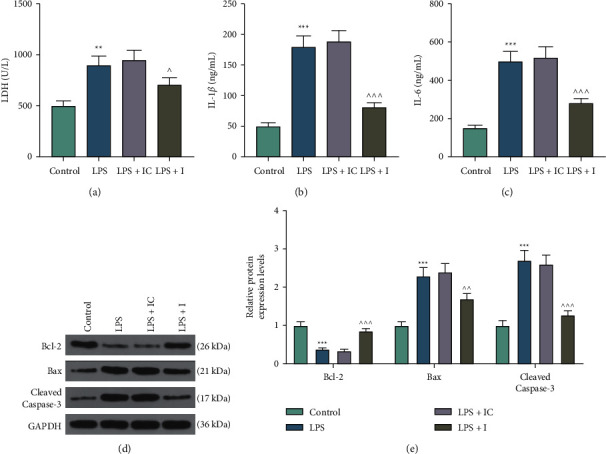
The downregulation of miR-197 suppressed the LPS-induced inflammation injury in H9C2 cells. (a) The LDH assay kit was applied to measure the level of LDH in LPS-induced H9c2 cells transfected with or without the miR-197 inhibitor. (b, c) ELISA was conducted to evaluate the proinflammatory factors, IL-1*β* and IL-6. (d, e) The protein levels of apoptosis-related genes (Bcl-2, Bax, and cleaved caspase-3) were tested using western blot. GAPDH was used as the loading control. The experiments were repeated three times. ^*∗∗*^*p* < 0.01; ^*∗∗∗*^*p* < 0.001 vs. control; ^∧^*p* < 0.05; ^∧∧^*p* < 0.01; ^∧∧∧^*p* < 0.001 vs. LPS + IC. LDH, lactate dehydrogenase; ELISA, enzyme-linked immunosorbent assay; LPS, lipopolysaccharide; IL-1*β*, interleukin-1*β*; IL-6, interleukin-6; Bcl-2, B-cell lymphoma-2; Bax, BCL2-associated X; GAPDH, glyceraldehyde-3-phosphate dehydrogenase; I, miR-197 inhibitor; IC, inhibitor control.

**Figure 3:**
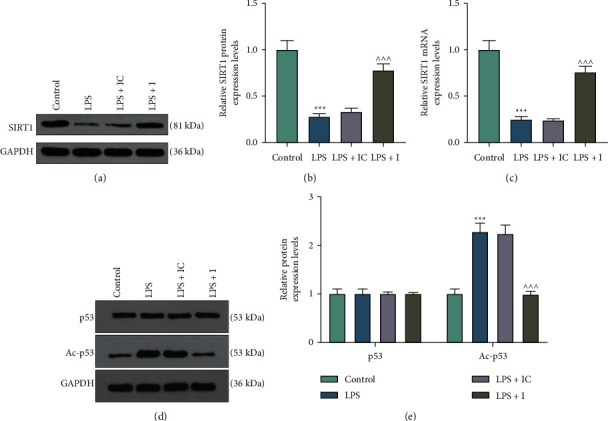
Effects of miR-197 inhibition on the expressions of SIRT1 and p35 in LPS-induced H9C2 cells. (a, b) Western blot was performed to measure the protein level of SIRT1 in LPS-induced H9C2 cells transfected with or without the miR-197 inhibitor. GAPDH was used as the loading control. (c) The mRNA expression of SIRT1 was then determined by qRT-PCR. GAPDH was used as the loading control. (d, e) The protein levels of p53 and AC-p53 in LPS-induced H9C2 cells transfected with or without the miR-197 inhibitor were evaluated via western blot. GAPDH was used as the loading control. ^*∗∗∗*^*p* < 0.001 vs. control; ^∧∧∧^*p* < 0.001 vs. LPS + IC. SIRT1, silent information regulator 1; LPS, lipopolysaccharide; qRT-PCR, quantitative real-time polymerase chain reaction; AC-p53, acetyl-p53; GAPDH, glyceraldehyde-3-phosphate dehydrogenase; I, miR-197 inhibitor; IC, inhibitor control.

After cell transfection, we evaluated the expression of miR-197 in the LPS-induced H9c2 cells. As a result, the overexpression of miR-197 induced by LPS was reversed by miR-197 inhibitor (*p* < 0.001) (Figure 1(c)). Cell functional experiments revealed that LPS significantly inhibited viability but promoted apoptosis of H9c2 cells as compared with the control group, and these LPS-induced effects were partially reversed by the miR-197 inhibitor (*p* < 0.01) (Figures 1(d)–1(f)). As demonstrated in Figure 2(a), a strike increase of LDH level was observed in the LPS group compared with that in the control group, and this trend was partially reversed by the miR-197 inhibitor (*p* < 0.05). In addition, the results of ELISA indicated that LPS remarkably elevated the levels of IL-1*β* and IL-6 in H9c2 cells, which were evidently restored by the miR-197 inhibitor (*p* < 0.001) (Figures 2(b) and 2(c)). Moreover, we applied western blot to measure apoptosis-related biomarkers in LPS-induced H9c2 cells transfected with or without the miR-197 inhibitor. Compared with the control group, Bcl-2 expression was reduced, while Bax and cleaved caspase-3 expressions were increased in the LPS group, which were partially reversed by miR-197 inhibitor (*p* < 0.01) (Figures 2(d) and 2(e)). Based on a previous study identifying the pivotal role of SIRT1 in protecting cardiomyocytes from oxidative stress injury [24], we subsequently determined the expression of SIRT1 in H9c2 cells. Western blot and qRT-PCR revealed that LPS notably downregulated SIRT1 expression, which was reversed via miR-197 inhibition (*p* < 0.001) (Figures 3(a)–3(c)). Furthermore, a considerable increase in AC-P53 protein level was observed in the LPS group, and this tendency was overtly countervailed in the LPS + I group (*p* < 0.001) (Figures 3(d) and 3(e)).

### 3.3. miR-197 Participated in LPS-Induced Cardiomyocyte Injury by Modulating SIRT1

**Figure 4:**
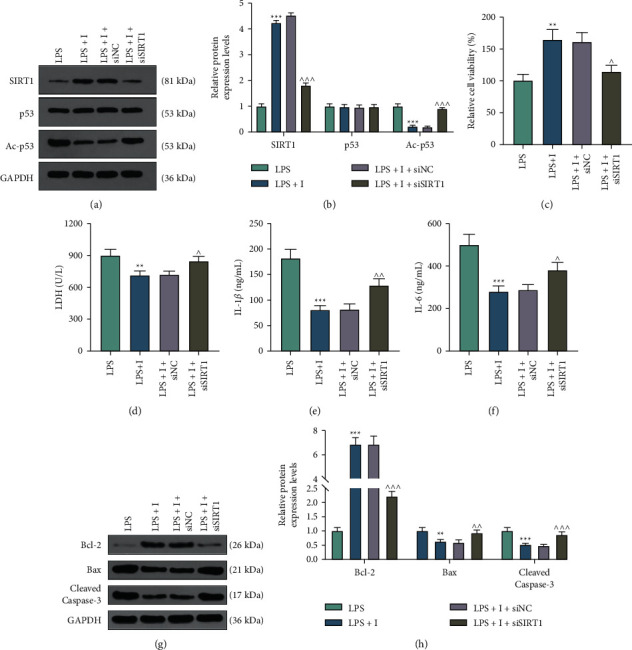
miR-197 participated in LPS-induced cardiomyocyte injury by modulating SIRT1. (a, b) To affirm the effect of transfection with siSIRT1, a western blot was conducted to measure the protein level of SIRT1. GAPDH was used as the loading control. (c) CCK-8 was used to examine cell viability. (d) After transfection with siSIRT1, the LDH assay kit was employed to measure levels of LDH. (e, f) ELISA was applied for evaluating IL-1*β* and IL-6 levels. (g, h) Western blot was utilized to examine the protein levels of apoptosis-related genes Bcl-2, Bax, and cleaved caspase-3. The abovementioned experiments were repeated at least three times. ^*∗∗*^*p* < 0.01; ^*∗∗∗*^*p* < 0.001 vs. LPS; ^∧^*p* < 0.05; ^∧∧^*p* < 0.01; ^∧∧∧^*p* < 0.001 vs. LPS + I + siNC. CCK-8, cell counting kit-8; qRT-PCR, quantitative real-time polymerase chain reaction; LDH, lactate dehydrogenase; ELISA, enzyme-linked immunosorbent assay; SIRT1, silent information regulator 1; LPS, lipopolysaccharide; IL-1*β*, interleukin-1*β*; IL-6, interleukin-6; Bcl-2, B-cell lymphoma-2; Bax, BCL2-associated X; GAPDH, glyceraldehyde-3-phosphate dehydrogenase; siSIRT1, small interfering RNA against SIRT1; I, miR-197 inhibitor; IC, inhibitor control; NC, negative control.

We then transfected H9c2 cells with siSIRT1 in order to further explore the interaction between miR-197 and SIRT1 under the induction of LPS. As illustrated in Figures 4(a) and 4(b), western blot verified an inhibitory effect of siSIRT1 on the expression of SIRT1 in the LPS + I + siSIRT1 group when compared with the LPS + I + siNC group (*p* < 0.001). In the CCK-8 assay, the promoting role of the miR-197 inhibitor in cell viability was visibly offset by siSIRT1 (*p* < 0.05) (Figure 4(c)). Besides, an increased amount of LDH release was observed in the LPS + I + siSIRT1 group compared with that in the LPS + I + siNC group (*p* < 0.05) (Figure 4(d)). Furthermore, siSIRT1 promoted IL-1*β* and IL-6 levels and reversed the effects of the miR-197 inhibitor (*p* < 0.05) (Figures 4(e) and 4(f)). Moreover, Bcl-2 expression was decreased, but the expressions of Bax and cleaved caspase-3 were increased in the LPS + I + siSIRT1 group when compared with those in the LPS + I + siNC group, indicating that siSIRT1 reversed the regulatory effect of the miR-197 inhibitor on apoptosis-related biomarkers (*p* < 0.01) (Figures 4(g) and 4(h)).

## 4. Discussion

Sepsis-induced cardiomyopathy occurs in about 40% of patients with sepsis, based on a deeper understanding of the pathology of sepsis and analysis of clinical big data [25]. Without effective treatments, patients with this disease may have an explicitly increased risk of death. In this study, LPS-induced H9c2 cells were used to mimic sepsis-induced cardiomyopathy injury *in vitro*. We observed the effect of miR-197 on the pathophysiological mechanism of LPS-induced H9c2 cells through *in vitro* experiments and found that miR-197 level was markedly upregulated, and the downregulation of miR-197 strikingly attenuated LPS-induced injury of H9c2 cells by upregulating the expression of SIRT1.

A study identified and evaluated prognostic miRNAs in the serum of patients with inflammatory heart disease and found that miR-197 could be used to distinguish between patients with viral or inflammatory disease and healthy donors, with a specificity of over 93% [26]. Meanwhile, it was manifested that platelet-enriched miR-197 was reduced in endotoxaemic volunteers receiving antiplatelet therapy [27]. Based on the abovementioned findings, the upregulation of miR-197 in LPS-induced H9c2 cells was found in our study, indicating that miR-197 might be implicated in sepsis-induced myocarditis. Yao et al. have found increased toxicity and apoptosis in H9c2 cells after LPS treatment, which is consistent with our results [28]. Through cell transfection, we found that miR-197 inhibition could reverse the effect of LPS, which prompted us to further investigate the mechanism of miR-197 in LPS-induced myocardial injury.

Currently, it is thought that a dysfunctional inflammatory response is the predominant mechanism of myocardial damage caused by sepsis [29]. Studies have shown that during the development of sepsis, inflammatory cytokines IL-1*β* and IL-6 work in concert to initiate the inflammatory response and have a negative inotropic effect on the myocardium [30–32]. Upregulation of IL-6 could reduce the action potential duration of cardiomyocytes, decrease the peak calcium transient, and diminish the release of Ca^2+^ from the sarcoplasmic reticulum [33]. In addition, upregulation of IL-1*β* could also decrease the calcium inward flow to cardiomyocytes, resulting in impairment of myocardial contractility [34]. This study demonstrated that miR-197 inhibitor significantly suppressed the upregulations of IL-1*β* and IL-6 in H9c2 cells under LPS treatment, suggesting that miR-197 could regulate inflammatory responses to mediate LPS-induced myocardial injury.

Additionally, continuous inflammation will lead to matrix degradation and apoptosis of cardiomyocytes [35, 36]. During cell apoptosis, Bax and cleaved caspase-3 are recognized as proapoptotic molecules, and Bcl-2 plays a key role in the regulation of mitochondrial permeability [28]. In our study, the results of western blot revealed that Bcl-2 level was distinctly decreased, and Bax and cleaved caspase-3 levels were overtly increased in LSP-induced H9c2 cells, suggesting enhanced apoptosis of cardiomyocytes. Furthermore, the present study found that downregulation of miR-197 reversed the expression of apoptosis-related proteins in LPS-treated H9c2 cells, and reduced the apoptosis of cardiomyocytes.

SIRT1 has been verified to be related with apoptosis and inflammation in cardiomyocytes and is also a key target for intervention therapy of alleviating myocardial injury caused by sepsis [37]. Rane et al. have revealed that miR-199a could regulate the metabolism of cardiomyocytes through SIRT1 [38]. Subsequently, the study by Zhu et al. has indicated that upregulation of miR-195 expression could promote the apoptosis of cardiomyocytes by inhibiting the SIRT1 level [39]. The abovementioned reports signify that the intervention of SIRT1 in myocardial injury might be regulated by multiple miRNAs. In this study, we identified that SIRT1 expression in LPS-treated cardiomyocytes could be regulated by miR-197. This finding suggests that the role of miR-197 in myocardial injury may be achieved by modulating the expression of SIRT1. Evidence has demonstrated that p53 is an important gene associated with apoptosis, senescence, and gene repair, playing an important role in myocardial injury [40]. p53 is a nonhistone substrate of SIRT1, and upregulation of p53 activates transcription of the target gene p21, which blocks the cell cycle in the G1/S phase, thereby inhibiting cell proliferation and further promoting cellular senescence [41]. Enhanced deacetylation of p53 by SIRT1 has been found to inhibit the activity of p53 and delay cellular aging [42]. Consistently, this study revealed that downregulation of miR-197 could inhibit p53 deacetylation in H9c2 cells under LPS induction. Based on these findings, our rescue assays indicated that the protective role of miR-197 downregulation in LPS-induced H9c2 cells was reversed by SIRT1 silencing.

## 5. Conclusion

In conclusion, the present study proved that miR-197 contributed to lipopolysaccharide-induced cardiomyocyte injury by modulating SIRT1. The present study provides a novel insight into the pathogenesis of myocarditis by focusing on the regulation of miR-197 under sepsis induction. However, the regulatory mechanism of miR-197 in sepsis-induced myocardial injury *in vivo* is not investigated in the current study, which will be improved in our future studies.

## Data Availability

The analyzed data sets generated during the study are available from the corresponding author on reasonable request.
